# Nondestructive Characterization of Internal Defects in Fiber Couplers Using OFDR-Based Thermal Excitation

**DOI:** 10.3390/s26175466

**Published:** 2026-08-29

**Authors:** Hao Lin, Mingye Fu, Xiang Zhang, Cuofu Lin, Jun Yang

**Affiliations:** 1 School of Information Engineering, Guangdong University of Technology, Guangzhou 510006, China; 2 Institute of Advanced Photonics Technology, School of Information Engineering, Guangdong University of Technology, Guangzhou 510006, China; 3 Key Laboratory of Photonic Technology for Integrated Sensing and Communication, Ministry of Education of China, Guangdong University of Technology, Guangzhou 510006, China; 4 Guangdong Provincial Key Laboratory of Information Photonics Technology, Guangdong University of Technology, Guangzhou 510006, China

**Keywords:** optical frequency-domain reflectometry, thermal excitation, fiber coupler, distributed strain, thermomechanical non-uniformity, nondestructive screening

## Abstract

Fiber-optic devices are widely used in communication, sensing, and other fiber-optic systems, and their quality directly affects system performance and reliability. However, internal defects in packaged devices remain difficult to characterize nondestructively, limiting their application in extreme environments. This work presents an active thermal-excitation method that uses the fiber embedded within the device as a distributed sensor. Controlled temperature changes are applied to excite the thermal response of the internal structure. Optical frequency-domain reflectometry (OFDR) then measures the distributed thermal strain along the fiber at millimeter-scale spatial resolution, enabling characterization of otherwise invisible internal defects. Using fused-fiber couplers as representative devices, we evaluated non-uniformity defects in the fused-taper and package-bonding regions. A finite-element model of an idealized coupler under thermal excitation was established to define the ideal thermal-response characteristics. The experimental results were broadly consistent with the simulated responses. Deviations from the predicted bonding-region symmetry and fused-taper uniformity indicated the possible presence of package-bonding and fused-taper defects, respectively. Dismantling representative couplers confirmed the locations and types of the defects. Applying the same criteria to the tested 2 × 2 and 1 × 3 couplers supported the applicability of the method across configurations within the present sample set. This method provides a transferable, high-spatial-resolution, and nondestructive means for quality assessment and reliability screening of packaged fiber-optic devices.

## 1. Introduction

Fiber-optic devices are key components of optical communication, sensing, and measurement systems, including systems deployed in harsh environments. Their compact size and compatibility with fiber links support broad system integration [1,2,3]. In practical applications, however, insufficient reliability under high- or low-temperature conditions can cause performance degradation or device failure, particularly in outdoor and other extreme environments. Manufacturing and packaging variations are difficult to observe after sealing, but they can produce stress concentrations, asymmetric constraints, and non-uniform strain transfer during temperature changes. Nondestructive screening of these hidden thermomechanical irregularities is therefore required to identify high-risk devices before deployment.

Taking fused-fiber couplers, which are among the most widely used fiber-optic devices, as an example, their long-term reliability and stability are strongly affected by fused-taper non-uniformity introduced during fabrication and package-bonding non-uniformity introduced during assembly [4,5]. These two process-related defects directly affect the functional coupling region and the principal load-transfer boundaries and may lead to performance degradation or device failure. Effective defect screening is therefore needed to reject high-risk and low-quality couplers before deployment.

Existing OFDR-based inspection and characterization methods mainly analyze Rayleigh backscattering, reflection, or transmission responses to obtain optical parameters such as propagation loss, insertion loss, return loss, reflectance, polarization, and coupling characteristics. Alegria and Zervas developed a nondestructive technique for characterizing coupler parameters from optical measurements [6]. Soller et al. used polarization-sensitive high-resolution OFDR to characterize optical components and assemblies, enabling the spatial localization of internal optical features [7]. Tokushima and Ushida applied OFDR backscattering measurements to characterize the transmission and reflection responses of waveguides and grating couplers in silicon photonic circuits [8]. Ponomarev et al. recorded OFDR reflectograms of proton-exchange channel waveguides during thermal cycling and localized temperature-induced changes in back reflection and optical loss [9]. However, these studies primarily analyzed optical-response parameters rather than using the thermally induced strain field of an internal fiber to characterize package-related structural defects.

Other structural inspection methods also have limitations. Ultrasonic imaging can provide information on internal fiber damage, but its spatial resolution may be insufficient for millimeter-scale defects in packaged fiber-optic devices [10]. X-ray computational tomography can visualize internal structures, but its cost and accessibility limit its use in routine screening, while X-ray exposure may also affect radiation-sensitive optical fibers [11,12]. Therefore, an inspection method that can nondestructively characterize the internal thermomechanical response of a fully packaged device is still needed. In contrast to conventional optical-parameter measurements, the present method uses the internal fiber as a distributed mechanical sensing element and combines active thermal excitation with OFDR strain measurements to reveal package-bonding asymmetry and fused-taper non-uniformity that may not yet produce detectable changes in the overall optical response.

To address this limitation, we propose an OFDR-based active thermal-excitation test for packaged fiber couplers that exploits the high spatial resolution of OFDR to measure the distributed thermally induced strain along the internal fiber. A sequence of temperature steps is applied within the rated operating range, and a reference fiber is used to remove the direct-temperature response, leaving the additional mechanical strain transferred by the package. An idealized symmetric finite-element model defines the normal response baseline. Subsequently, package-bonding and fused-taper defects are evaluated from the symmetry between the two end-connection regions and the spatial uniformity of the central fused-taper region, respectively. A 1 × 3 coupler is analyzed in detail, and four additional couplers (two 2 × 2 and two 1 × 3 devices) are used to evaluate the applicability of the screening procedure. The method is applicable to devices containing a continuous fiber or waveguide path that can be interrogated by OFDR; it does not detect every internal defect or uniquely determine its manufacturing cause.

## 2. Method and Thermomechanical Reference

### 2.1. Coupler Structure and Principal Inspection Targets

**Figure 1:**
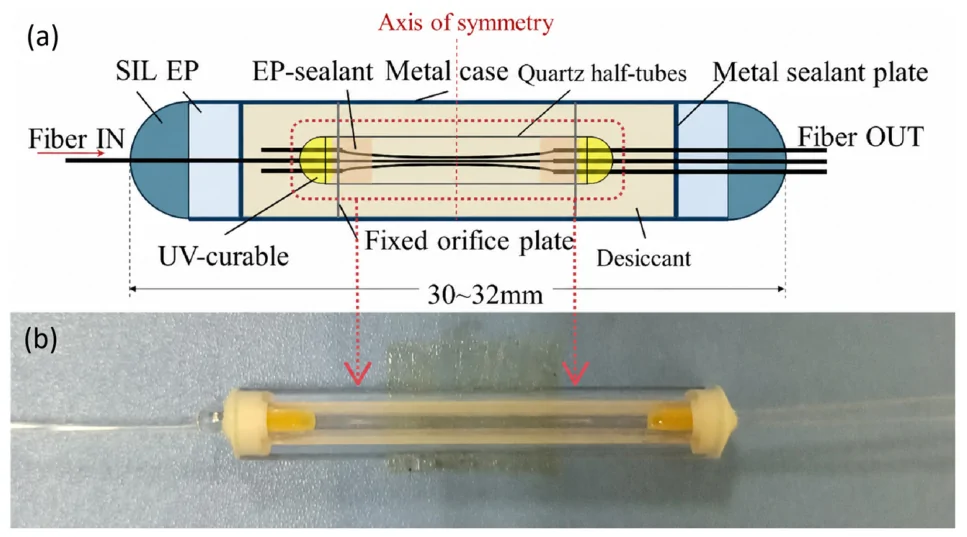
Structure of the packaged 1 × 3 fused-fiber coupler: (**a**) schematic showing the central fused-taper section, the two end-bonding regions, the support/fixing components, and the nominal symmetry axis; (**b**) photograph of the central assembly before final closure.

A packaged 1 × 3 fused-fiber coupler was used as the representative specimen. As shown in Figure 1a, the package consists of input/output fiber pigtails, a central fused-taper functional section, and a quartz half-tube support. It also includes two bonded transition regions, fixing plates and an outer metal housing. The bonded transition regions connect the slender functional fiber to the support and housing, thereby defining the primary boundaries for thermomechanical load transfer.

The central fused-taper section is formed by bringing three single-mode fibers into contact and then locally heating, pulling, and fusing them into a composite waveguide. Before tapering, the fibers have a cladding diameter of approximately 80 µm and a core diameter of 8–10 µm. The resulting diameter reduction increases optical-field overlap and enables power transfer between adjacent cores, but it also decreases the local axial stiffness. Recent multicore-coupler research likewise shows that the fiber arrangement and interaction region determine the resulting coupling behavior [13]. For the specimen shown in Figure 1a, the package length is approximately 30–32 mm, whereas the quartz-supported central section is approximately 14 mm long. The transition and sealing regions contain silicone rubber, epoxy resin, UV-curable adhesive, and epoxy sealant. Figure 1b shows the internal assembly before closure of the outer metal housing.

Manufacturing and packaging defects in fused-fiber couplers include incorrect pulling length, a non-uniform waist diameter, insufficient or excessive fusion, fiber misalignment, surface microcracks, uneven adhesive deposition, incomplete curing, internal voids, and residual assembly stress. This study focuses on fused-taper non-uniformity and package-bonding non-uniformity because these defect classes directly affect the optical coupling region and the primary thermomechanical load-transfer boundaries, respectively. Severe defects can produce excess loss, coupling-ratio drift, cracking, or fiber fracture and ultimately lead to functional failure.

Fused-taper non-uniformity refers to unintended axial or transverse variations in the waist diameter, taper length, transition geometry, or degree of fusion. Such non-uniformity can arise from non-uniform heating, variations in pulling speed or axial tension, or fiber misalignment. Insufficient pulling or an excessively short taper may prevent the designed optical coupling condition from being reached. Excessive pulling can instead produce an overly thin waist, reducing its load-bearing margin and potentially compromising long-term reliability [14]. A recent review of fused-tapered couplers summarized the effects of taper geometry and fabrication conditions on coupling and sensing performance [15]. Experimental characterization of a fluoride-fiber coupler further demonstrated the influence of material selection and taper fabrication on transmission loss and coupling performance [16]. These geometric, material, and fusion variations are therefore expected to generate localized peaks, troughs, or fluctuations in the thermal-strain profile of the central fused-taper section.

Package-bonding non-uniformity refers to differences between the two bonded ends in bonded length, adhesive thickness, bonding position, effective stiffness, cure state, or void distribution. Such non-uniformity can arise from inconsistent dispensing, asymmetric positioning, curing shrinkage, or thermal-expansion mismatch. These differences can produce asymmetric load transfer and local stress concentration near the bond boundaries, thereby increasing the risk of performance drift, crack initiation, and fiber breakage [14]. Accordingly, this asymmetry is expected to produce systematic differences between the temperature-dependent responses of the left and right bonded regions.

The resulting practical inspection targets were (i) the spatial uniformity of the central fused-taper response and (ii) the symmetry of the two end-bonding responses. A nominally conforming device should exhibit a comparatively smooth central profile and similar responses at geometrically matched positions near the two ends. These response-based criteria are used for screening; they indicate thermomechanical non-uniformity but cannot, by themselves, uniquely identify every underlying manufacturing cause.

The required inspection scale is determined by the dimensions of the coupler. Specifically, the package is 30–32 mm long, the central fused-taper section is about 14 mm long, and each bonded transition spans only several millimeters. Resolving local fluctuations within the taper and distinguishing between the two end responses therefore require distributed measurements with millimeter-scale spatial resolution and microstrain-level sensitivity.

### 2.2. Active Thermal-Excitation Measurement Principle

To screen the two defect classes, we propose an active thermal-excitation method that uses the embedded fiber as a distributed sensing element. A controlled temperature change excites the internal thermomechanical response, and the resulting distributed axial strain is read along the fiber. Temperature-dependent coupler experiments have shown that thermal expansion and thermally induced alignment variations can alter the coupling response [17]. Distributed OFDR strain measurements have also been used to localize stress concentrations associated with structural defects [18], supporting the use of distributed strain profiles for spatial screening. The temperature change acts as a known stimulus, while local variations in geometry or mechanical constraint modulate the resulting strain field and generate spatial contrast. Unlike passive end-to-end optical testing, the distributed strain profile provides spatial information about internal load transfer rather than only an aggregate device response.

For a homogeneous and unconstrained material, a temperature change Δ*T* produces the free thermal strain $\varepsilon_{t}$ defined by Equation (1). When the fiber is bonded to materials with different thermal-expansion coefficients or restrained by fixed boundaries, its free thermal expansion is partially converted into mechanical stress and additional axial strain.

(1) $$\varepsilon_{t}=\alpha \cdot \Delta T$$

where $\varepsilon_{t}$ denotes the free thermal strain, *α* is the coefficient of thermal expansion, and Δ*T* is the temperature change relative to the reference state. In the packaged coupler, strain transferred to the fiber depends on local axial compliance and on the stiffness and geometry of the adjoining bonded materials. Controlled temperature variation can therefore render internal geometric and bonding differences detectable through their spatial strain signatures, even when they are not apparent at a single equilibrium temperature.

The measured OFDR spectral shift contains both the intrinsic temperature response of silica fiber and the mechanical strain transferred from the package. A free fiber subjected to the same temperature sequence provides the intrinsic thermal baseline. Subtracting that baseline from the packaged-fiber result gives the package-induced mechanical strain used in the subsequent regional analysis, as expressed by Equation (2).

(2) $$\varepsilon_{pkg}\left(x,T\right)=\varepsilon_{meas}(x,T)-\varepsilon_{free}(x,T)$$

where $\varepsilon_{pkg}\left(x,T\right)$ is the corrected package-induced axial strain, $\varepsilon_{meas}\left(x,T\right)$ is the OFDR-derived response of the fiber inside the coupler relative to the reference state, and $\varepsilon_{free}\left(x,T\right)$ is the representative response of the unconstrained reference fiber at the same temperature. The subsequent regional analysis uses $\varepsilon_{pkg}\left(x,T\right)$.

In the fused-taper region, the reduced fiber diameter and changed effective modulus increase local compliance. Under thermomechanical loading, strain can therefore concentrate in this central tapered region, potentially limiting device reliability. At the two ends, unequal bonded length, adhesive thickness, stiffness, or curing state changes the amount of load transferred into the fiber; the defect is therefore expressed as a persistent difference between responses at matched left and right positions. Thermal excitation does not create these defects; it converts their unequal constraints into a measurable strain contrast. The screening output is evaluated by region rather than reduced to a single end-to-end value. End-bonding symmetry is assessed from the difference between temperature-dependent responses at geometrically matched left and right feature points. Central fused-taper uniformity is assessed from the spatial standard deviation, peak-to-peak range, and peak-to-peak value normalized by the maximum absolute strain within the same window. Similar end responses and a comparatively smooth central profile define the normal reference; systematic departures flag the device for further inspection. Based on the coupler geometry described in Section 2.1, the minimum structural feature size is approximately 1.5–2 mm. Considering the trade-off between spatial resolution and measurement accuracy, OFDR was selected and operated at a spatial resolution of 2 mm. In the experimental system, a 2 mm spatial resolution is used so that the central window and the two end-bonding regions can be evaluated separately.

### 2.3. Finite-Element Reference Model

A finite-element model was constructed to establish the expected thermomechanical response of a nominally symmetric coupler. The material parameters are listed in Table 1. Representative elastic properties were used because complete temperature-dependent material data for the specific packaged device were unavailable.

The model was implemented in COMSOL Multiphysics 6.2 (COMSOL AB, Stockholm, Sweden) using the Solid Mechanics interface. A two-dimensional plane-stress, small-strain, quasi-static formulation represented the slender package as a bonded, heterogeneous assembly. Each domain was treated as homogeneous, isotropic, and linearly elastic, and the temperature was applied uniformly at discrete set points relative to 25 °C. For each material domain *i*, the mechanical equilibrium, strain–displacement relation, linear–elastic constitutive relation, and thermal strain were respectively written as follows:

(3) $$\nabla \cdot \sigma_{i}=0$$

(4) $$\varepsilon =\frac{\nabla u+{\left(\nabla u\right)}^{T}}{2}$$

(5) $$\sigma_{i}=C_{i}\left(E_{i},\nu_{i}\right):\left[\varepsilon -\varepsilon_{i}^{th}\right]$$

(6) $$\varepsilon_{i}^{th}=\alpha_{i}\left(T-T_{ref}\right)I$$

Here, u is displacement, ε is infinitesimal strain, $\sigma_{i}$ is stress in material *i*, $C_{i}$ is the elasticity tensor determined by $E_{i}$ and $\nu_{i}$, and $\alpha_{i}$ is the coefficient of thermal expansion. Bonded interfaces were continuous, the mounting boundaries were fixed, and the remaining exterior boundaries were traction-free. Axial strain was extracted along the fiber centerline.

For the one-dimensional axial interpretation, the corresponding thermoelastic constitutive relation can be expressed as:

(7) $$\sigma =E\left[\varepsilon_{tot}-\alpha \left(T-T_{ref}\right)\right]=E\varepsilon_{mech}$$

where $\varepsilon_{tot}$ is the total axial strain, $\alpha (T-T_{ref})$ is the free thermal strain, and $\varepsilon_{mech}$ is the mechanically constrained strain. This constitutive relation describes the thermoelastic stress calculation in the finite-element model and is not used for the experimental free-fiber baseline subtraction.

The geometry and constraints followed the observed package-scale load path. The metal housing, internal baffle, and quartz-tube fixing plates were treated as fixed supports, while the polymeric regions transferred the axial thermal load to the fiber. The model included approximately 2 mm outer silicone regions, approximately 2 mm epoxy regions, fixing plates about 4 mm from the seals, an approximately 14 mm quartz half-tube, approximately 1.5 mm external UV-curable adhesive regions, and approximately 3 mm internal epoxy sealant regions. These dimensions define the nominal symmetric reference and are not used to claim the exact adhesive geometry of the measured specimen.

Free triangular elements were refined around the fiber and adhesive interfaces. The solved mesh contained approximately 141,466 domain elements and 26,224 boundary elements. Because no systematic mesh convergence study or specimen-specific material characterization were available, predicted amplitudes are treated as estimates. Model experiment differences in absolute magnitude are not by themselves classified as defects; the model is used primarily to define the expected location, symmetry, and smoothness of the package response.

**Figure 2:**
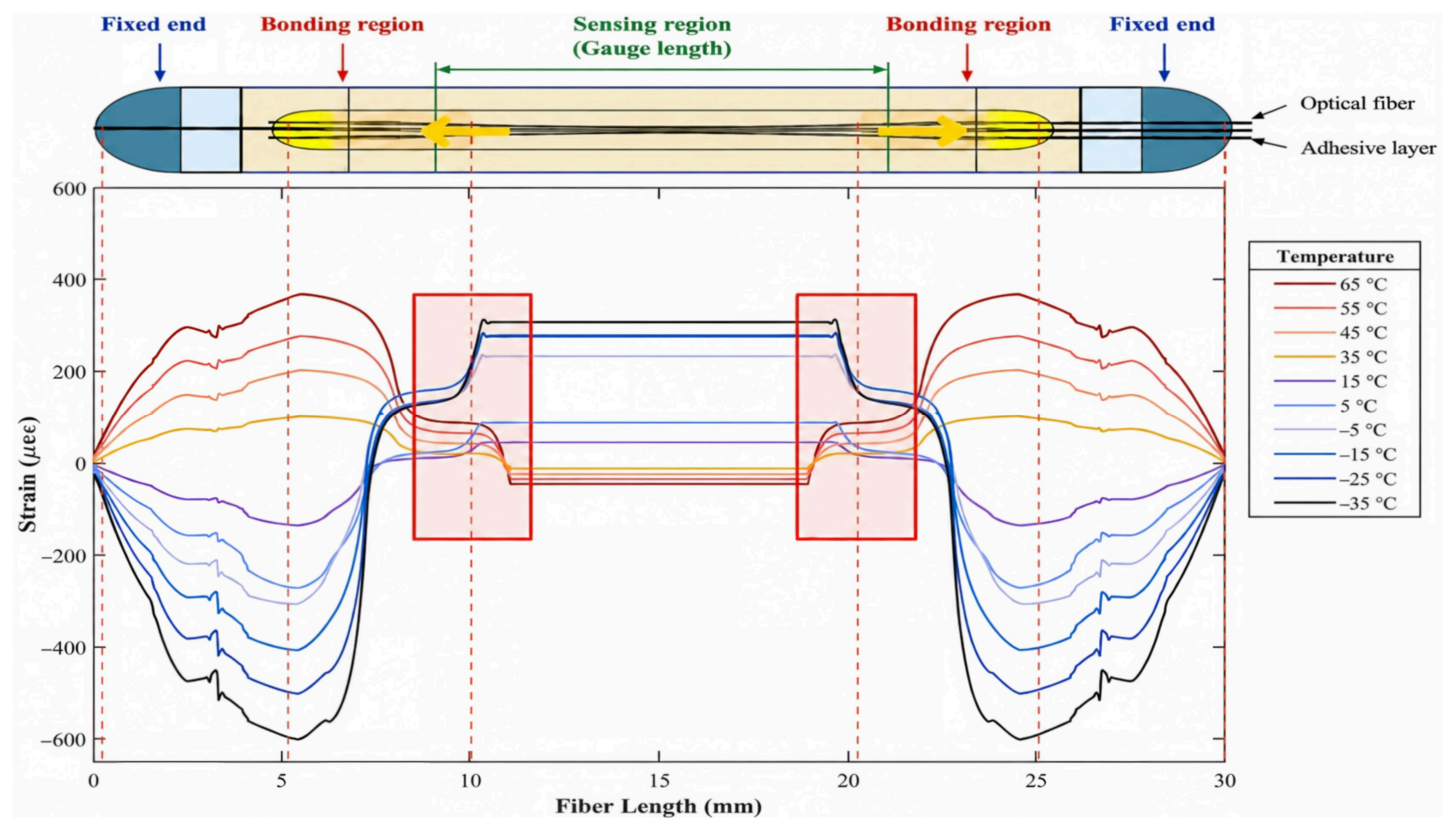
Schematic diagram of COMSOL simulation strain for coupler.

Figure 2 shows the axial-strain distribution calculated for the nominally symmetric package under thermal excitation. Load is introduced mainly through the two bonded transition regions. Because the idealized geometry, material assignment, and boundary conditions are mirror-symmetric, the left and right end extrema should have nearly identical magnitudes, and their strain–temperature curves should nearly coincide. Within each heating or cooling branch of the linear thermoelastic model, the response at a fixed position should also vary approximately linearly with temperature. In contrast, the ideal central fused-taper window should remain spatially smooth, without a repeatable local peak or trough. End-response asymmetry is therefore used to screen bonding non-uniformity, whereas abnormal spatial fluctuation in the central window is used to screen fused-taper non-uniformity.

**Figure 3:**
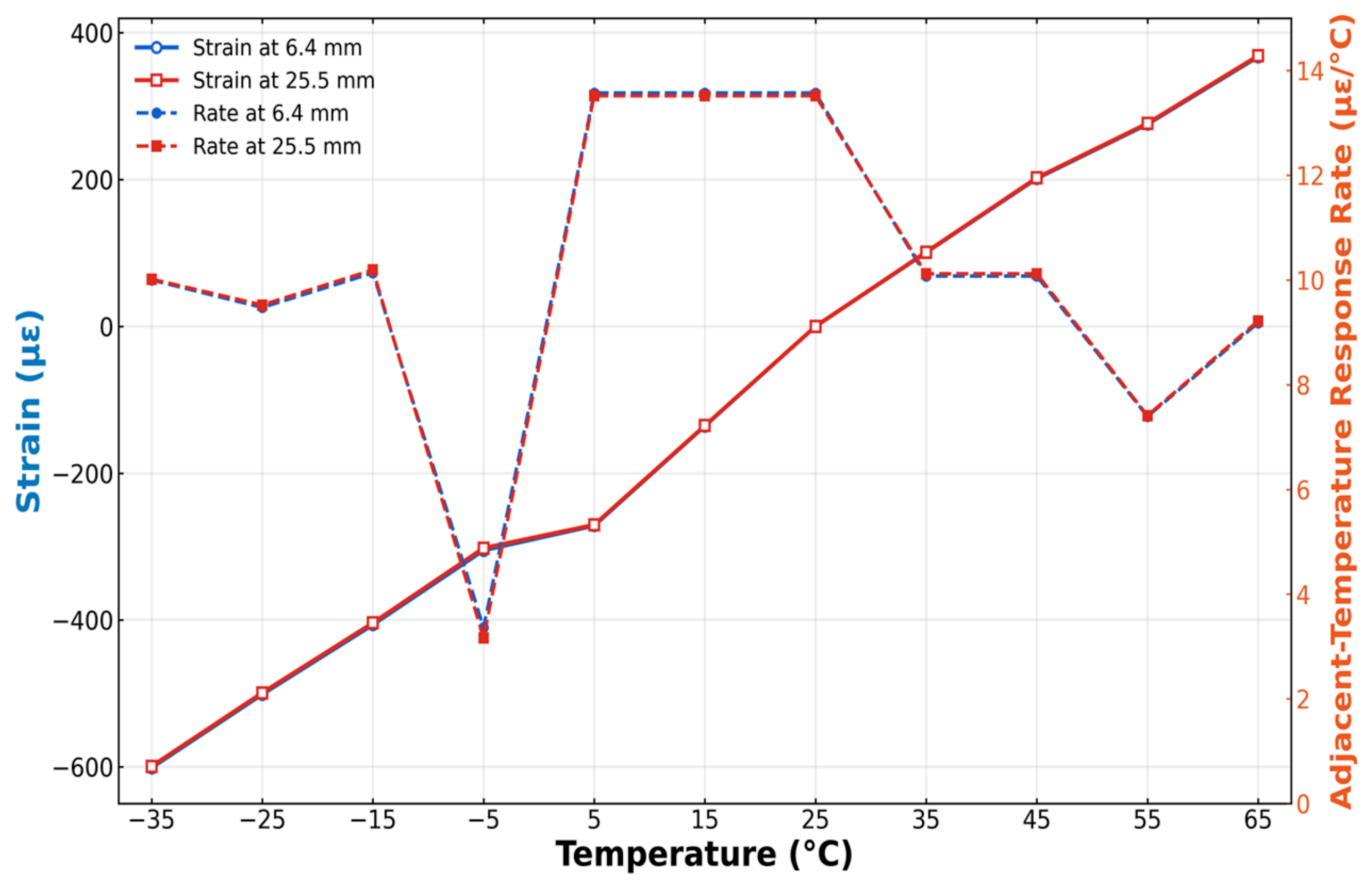
Simulated strain and adjacent-temperature response rate at the two fixed feature points (6.4 and 25.5 mm). Solid curves are strain values read from the left *y*-axis; dashed curves are adjacent-temperature finite-difference rates read from the right *y*-axis. The 25 °C state is the zero-strain reference and is not assigned an adjacent-temperature rate.

The two feature-point strain curves in Figure 3 nearly overlap over the full temperature sequence, confirming the left–right symmetry expected from the nominal model. The central profile in Figure 2 is likewise smooth compared with the localized end extrema. These two behaviors—rather than exact agreement at every amplitude—form the reference for interpreting the measured coupler. The near coincidence of the two rate curves remains the relevant result for the symmetry assessment.

## 3. Experiments and Results

### 3.1. Measurement System and Test Protocol

OFDR estimates the distributed axial-strain response by tracking the position-dependent spectral shift in the fiber’s intrinsic Rayleigh-scattering pattern. During measurement, a narrow-linewidth laser is swept continuously across a prescribed optical-frequency range, and its output is divided into reference and sensing paths. Light from the sensing path is launched into the coupler fiber, and the position-dependent Rayleigh-backscattered pattern interferes coherently with light from the reference path. After sweep calibration and Fourier-domain processing, the interference signal is mapped to distance along the fiber. At each fiber position, the local Rayleigh spectrum acquired at the excited temperature is cross-correlated with the corresponding spectrum recorded at the 25 °C reference state. The resulting position-dependent spectral shift is then converted into the axial-strain distribution. These operating principles and recent approaches for improving the spatial resolution, frequency-sweep linearity, signal-to-noise ratio, and strain/temperature demodulation have been systematically reviewed in [20].

**Figure 4:**
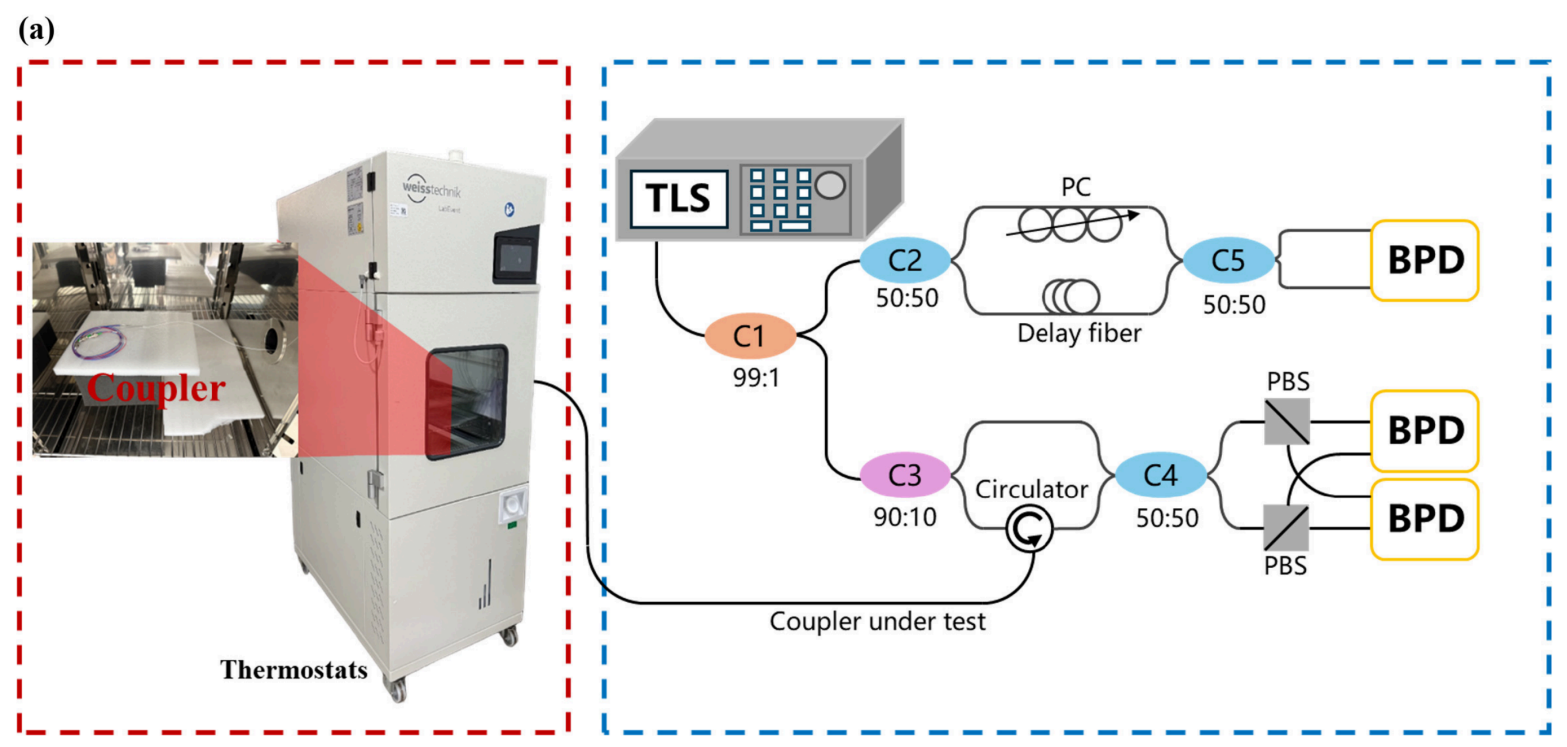

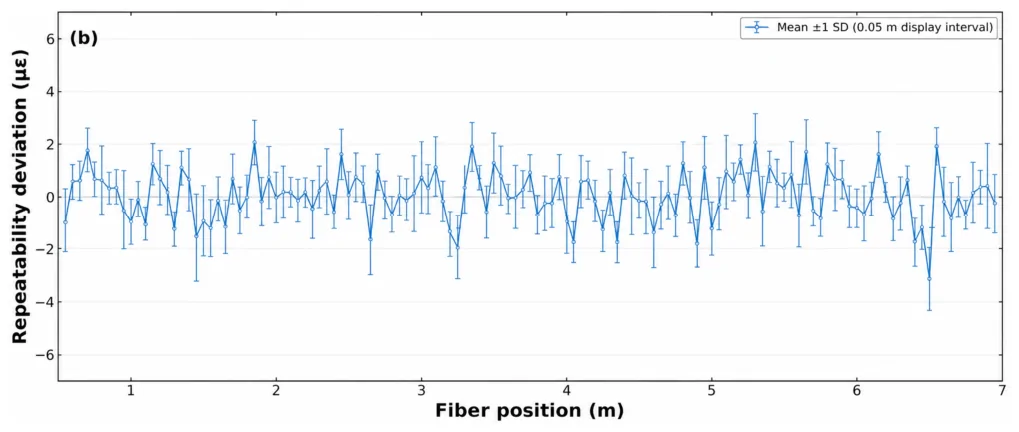
OFDR measurement system and short-term repeatability evaluation: (**a**) optical configuration of the OFDR-based thermal-excitation system for distributed strain measurement; (**b**) position-wise repeatability, shown as zero-centered ±1 standard deviation error bars at approximately 0.5 m intervals.

Figure 4a shows the measurement system, in which a tunable laser (TSL-770; Santec Corporation, Komaki, Aichi, Japan) was swept from 1480 to 1640 nm. A custom-built OFDR system comprising an auxiliary Michelson interferometer, a polarization-diverse Mach–Zehnder interferometer, balanced InGaAs detection (Nanjing Nanzhi Ruijie Optoelectronic Technology Co., Ltd., Nanjing, China), and synchronous 16-bit acquisition using a data-acquisition card (Beijing Qiyang Risheng Technology Co., Ltd., Beijing, China) was used.The common pigtail of the packaged 1 × 3 coupler was connected to the OFDR sensing port.The packaged coupler was placed in a programmable temperature chamber (Suzhou Guanghuan Technology Co., Ltd., Suzhou, China) for controlled thermal excitation.

For strain demodulation, a 2 mm spatial correlation window was translated along the fiber in 0.3 mm increments. The 2 mm window set the effective spatial resolution, whereas the 0.3 mm increment increased the sampling density and provided a more detailed thermal-strain profile. Because adjacent windows overlapped, the 0.3 mm output spacing did not represent an independent spatial resolution of 0.3 mm. As shown in Figure 4b, measurements repeated at 25 °C verified an OFDR strain measurement resolution of ±1.2 µε under the selected 2 mm demodulation setting [21]. The mean curve is not perfectly straight because small temperature variations along the fiber introduce minor spatial fluctuations in the measured strain. Experimental data processing and figure generation were performed using MATLAB R2023 (MathWorks, Natick, MA, USA).

**Figure 5:**
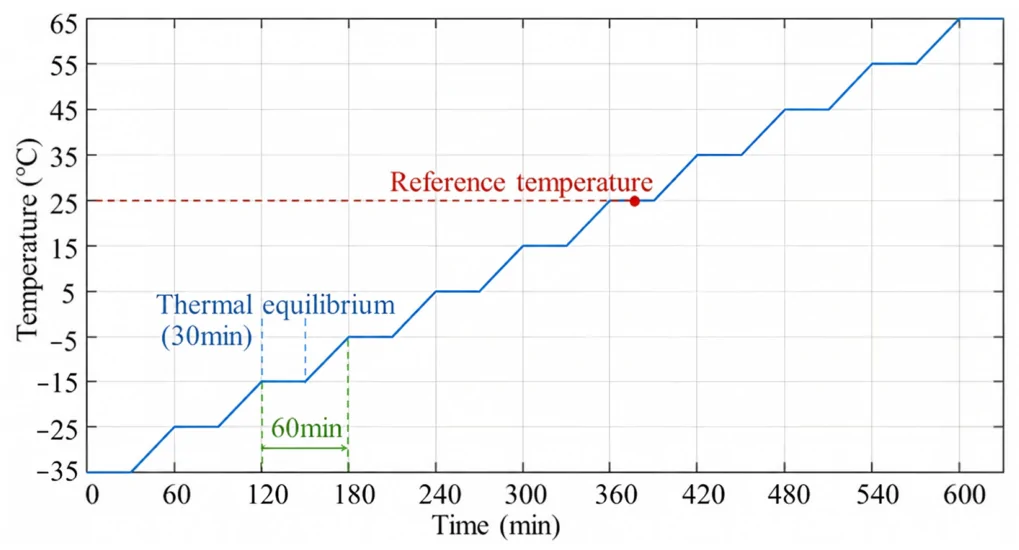
Thermal-excitation temperature-excitation curve.

The chamber temperature was stepped from −35 to 65 °C in 10 °C increments, and each setpoint was held for 30 min before the OFDR scan. As shown in Figure 5, all measurements were acquired during a single continuous heating sequence. The 25 °C scan served as the reference for differential strain, and all 10 non-reference temperature points were retained for regional analysis. The tested temperature range remained within the specified operating limits of the coupler, allowing its in-range thermomechanical response to be probed without intentionally inducing over-temperature damage.

### 3.2. Interrogation Path and Valid-Region Identification

**Figure 6:**
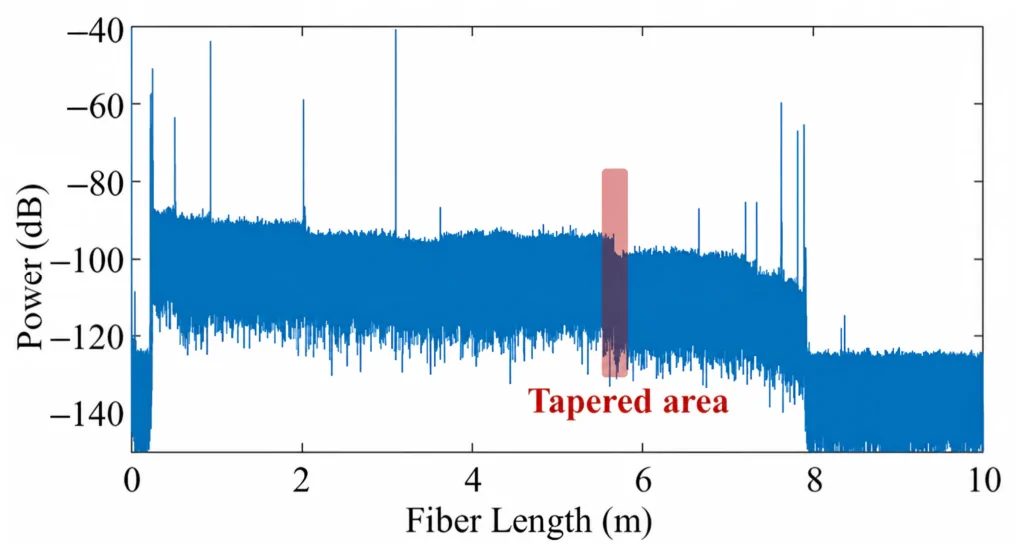
Forward-input Rayleigh reflection trace of the 1 × 3 single-mode fiber coupler.

The Rayleigh trace in Figure 6 located the connector interfaces and the packaged coupling section, with the V-shaped attenuation feature marking the fused-taper region. Using this feature, the structural analysis window was registered to the physical 0–30 mm package coordinate. The positive coordinate direction was defined from the input-fiber end toward the output-fiber end.

**Figure 7:**
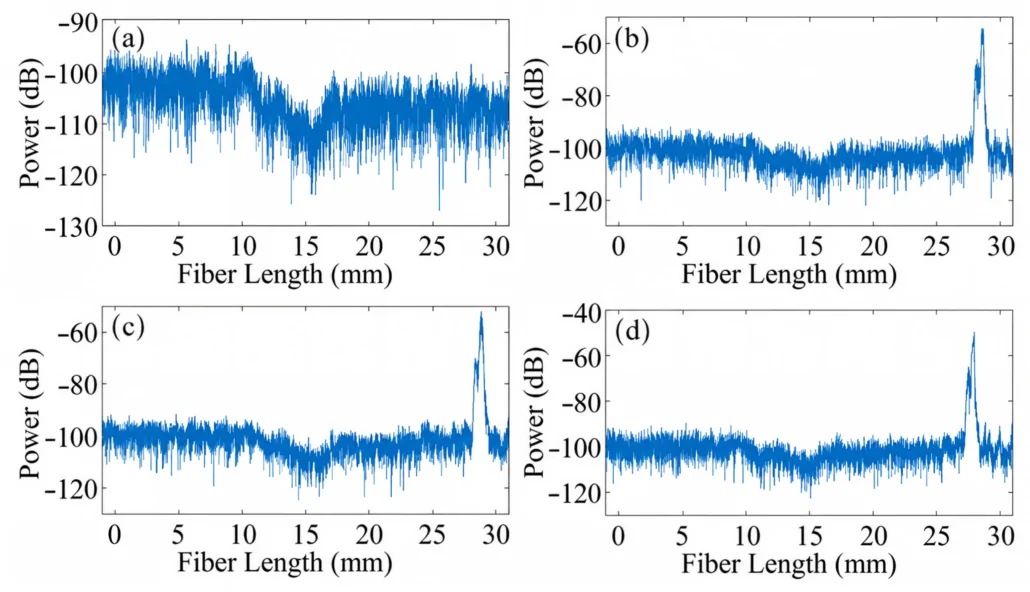
Reflection traces in the coupler package region: (**a**) forward test encapsulation area spectral details; (**b**) back tested Port 1 reflection trace; (**c**) back tested Port 2 reflection trace; (**d**) back tested Port 3 reflection trace.

Reverse interrogation produced two strong discrete reflection peaks associated with the terminated internal branches (Figure 7). Originally designed as a 3 × 3 device, the coupler was fabricated from three tapering fibers; however, two internal fibers were broken to form the measured 1 × 3 configuration. The high-reflection peaks were attributed to the exposed end faces of these fibers. These reflections can degrade correlation by consuming the local dynamic range or generating sidelobes. Accordingly, forward interrogation from the common port was used for the thermal-strain measurement. Although residual taper attenuation can lower the local signal-to-noise ratio (SNR), it does not represent physical strain, which is derived from spectral shifts rather than absolute amplitude. Low SNR can nevertheless increase correlation uncertainty and produce apparent strain errors.

### 3.3. Thermomechanical Screening Results

#### 3.3.1. Consistency Between the Reference Model and Measurement

**Figure 8:**
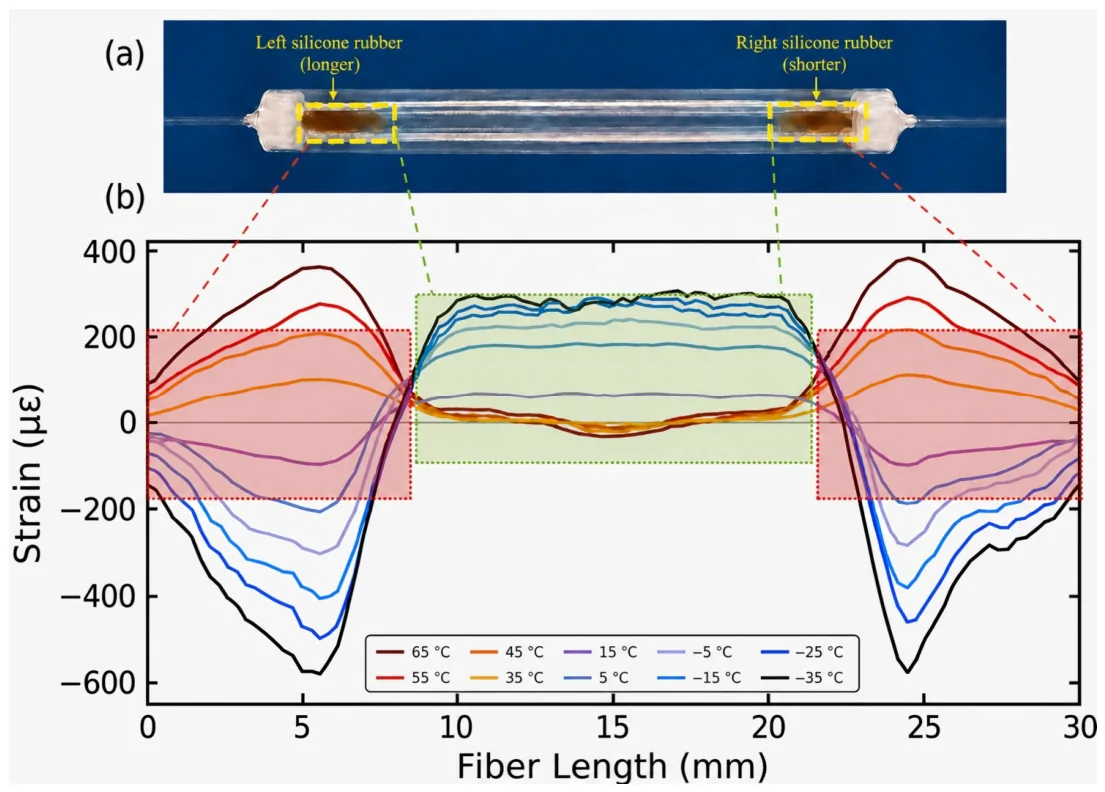
Experimental strain response and package geometry observation of the tested 1 × 3 fiber coupler: (**a**) photograph of the central taper region, showing the asymmetric effective geometries of the compliant silicone rubber and adhesive regions at the two ends; (**b**) distributed thermal-strain profiles measured by OFDR at −35, −25, −15, −5, 5, 15, 35, 45, 55, and 65 °C, relative to the 25 °C reference state.

Figure 8a shows the central assembly and effective geometries of the two bonded transition regions. Figure 8b shows the corrected package-induced strain profiles of the representative 1 × 3 coupler at the 10 non-reference temperatures, relative to 25 °C as the reference state.

Similarity between the simulation and experiment profiles was quantified using the metrics summarized in Table 2. Across the investigated temperatures, the full-profile correlation coefficients range from 0.936 to 0.982 (mean, 0.967), indicating strong similarity in the shapes of the simulated and measured strain profiles. Residual local differences may reflect idealized geometry, simplified material parameters, or numerical approximation in the simulation. The model was intended to define a reference response pattern rather than reproduce every local strain magnitude. Accordingly, subsequent analysis focused on the two prespecified screening features: response symmetry between the bonded transition regions and spatial uniformity within the central fused-taper section.

#### 3.3.2. Package Defect Analysis

**Figure 9:**
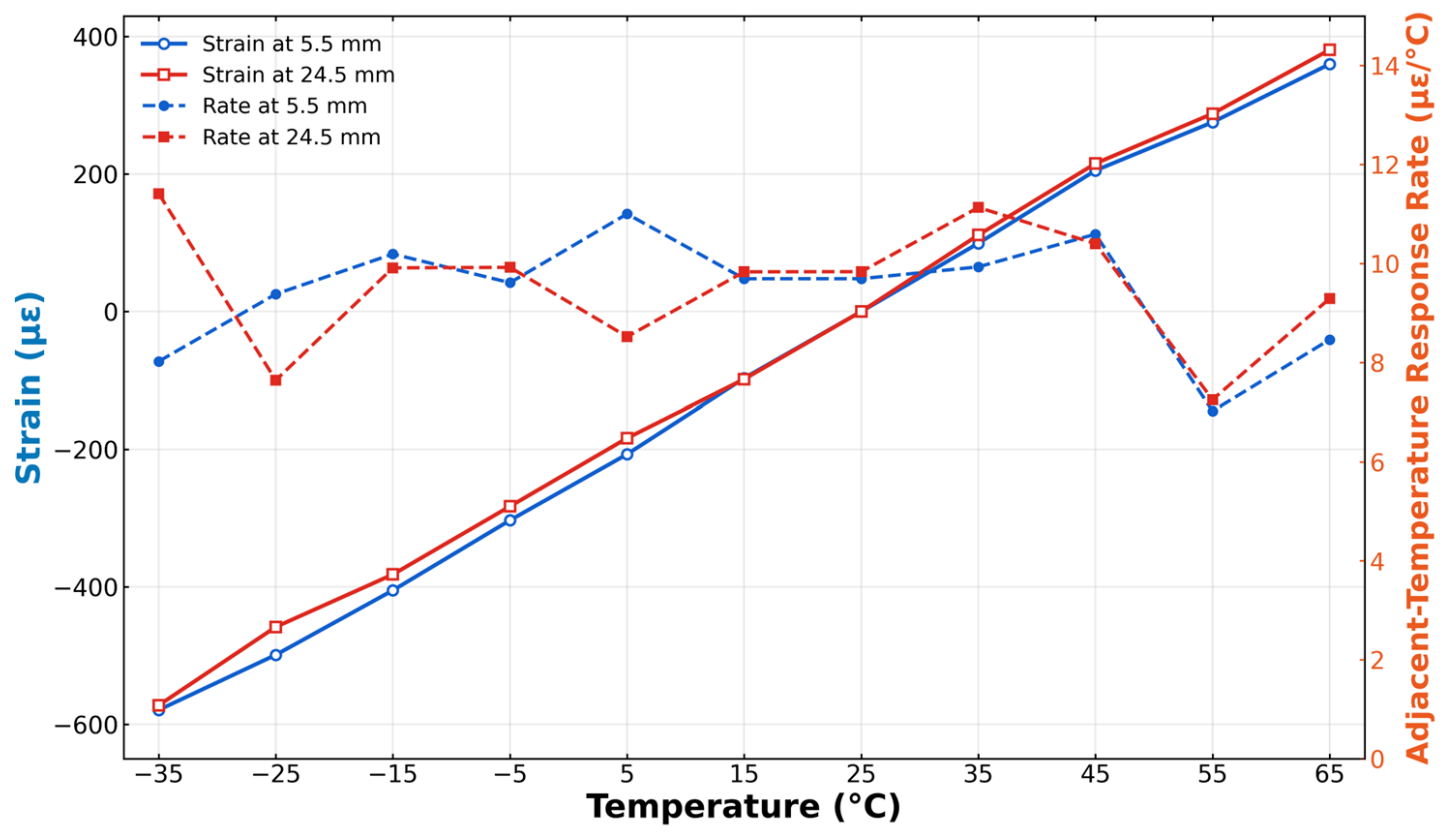
Experimental strain and adjacent-temperature response rate at the two fixed feature points (5.5 and 24.5 mm) across the tested temperature range. Solid lines indicate strain, and dashed lines indicate the finite-difference response rate between successive temperature set points; 25 °C is the zero-strain reference, for which an adjacent-temperature rate is not assigned.

Figure 9 compares the strain and adjacent-temperature response rates at matched feature points near the two bonded transition regions. The left and right point were located at 5.5 and 24.5 mm, respectively. The experimental curves followed the same overall temperature dependence as the ideal symmetric simulation in Figure 3. This similarity supports use of the model as a package-scale reference but does not establish local quantitative agreement.

As seen in Figure 9, the strain curves at the two end feature points remain close over the investigated temperature range, and their response rate curves showed the same similar overall variation. No pronounced or persistent separation is observed between the left and right responses. These patterns are consistent with broadly symmetric thermally induced load transfer between the bonded transition regions. Under the prespecified end-region symmetry criterion, the data provided no clear evidence of pronounced package-bonding non-uniformity, although smaller asymmetries cannot be excluded without a quantitative acceptance threshold.

#### 3.3.3. Fused-Taper Defects Analysis

**Figure 10:**
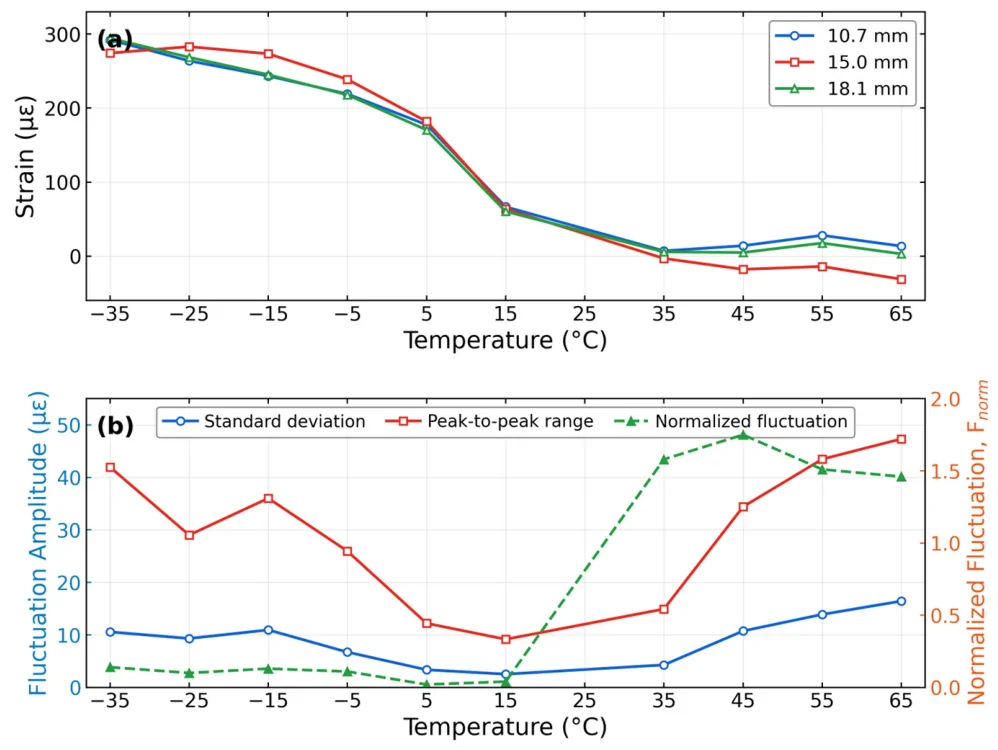
Temperature dependence of the experimental central-region response: (**a**) strain at three representative positions (10.7, 15.0, and 18.1 mm); (**b**) standard deviation and peak-to-peak range of the full central-region window (left axis), together with the normalized fluctuation index (right axis). Only the non-reference temperature conditions are shown because the normalized fluctuation index is undefined at the zero-strain reference state of 25 °C.

The central analysis window extended from 10.1 to 19.3 mm. Three representative positions at 10.7, 15.0, and 18.1 mm describe the observed peak–trough–peak pattern. To characterize the complete central window, the standard deviation, peak-to-peak range, and normalized fluctuation index were calculated across all 31 sampled points (Figure 10). Because adjacent 2 mm correlation windows overlapped by approximately 85%, these positions formed an oversampled spatial profile rather than 31 independent observations.

To account for changes in the overall strain magnitude, the peak-to-peak fluctuation was normalized by the maximum absolute strain in the same central region, as defined in Equation (8):

(8) $$F_{norm}=\frac{\varepsilon_{\max}-\varepsilon_{\min}}{\max\left|\varepsilon \right|}$$

The three positions followed the same overall temperature trend but remained separated in strain, unlike the smooth central response predicted by the symmetric model. The peak-to-peak range was 12.21 µε at 5 °C and 9.16 µε at 15 °C. Above the reference temperature, the central-window standard deviation increased from 4.28 µε at 35 °C to 16.44 µε at 65 °C. At elevated temperatures, a large normalized fluctuation index coincided with a small denominator in Equation (8) because the maximum absolute central-region strain approached zero. Thus, the normalized index was numerically amplified and should not be interpreted alone as evidence of large absolute fluctuations or reduced stability.

Within the range of 5–35 °C, both the spatial variation and peak strain of the coupler remained at their lowest levels. Under high-temperature conditions, the fused-taper region remained in a relaxed state and did not experience additional thermal strain. Under low-temperature conditions, the spatial variation and peak strain of the coupler deteriorated considerably. Based on the thermal-strain behavior described above, we speculate that the coupler may have a higher risk of failure at low temperatures.

### 3.4. Small-Batch Coupler Test Validation

**Figure 11:**
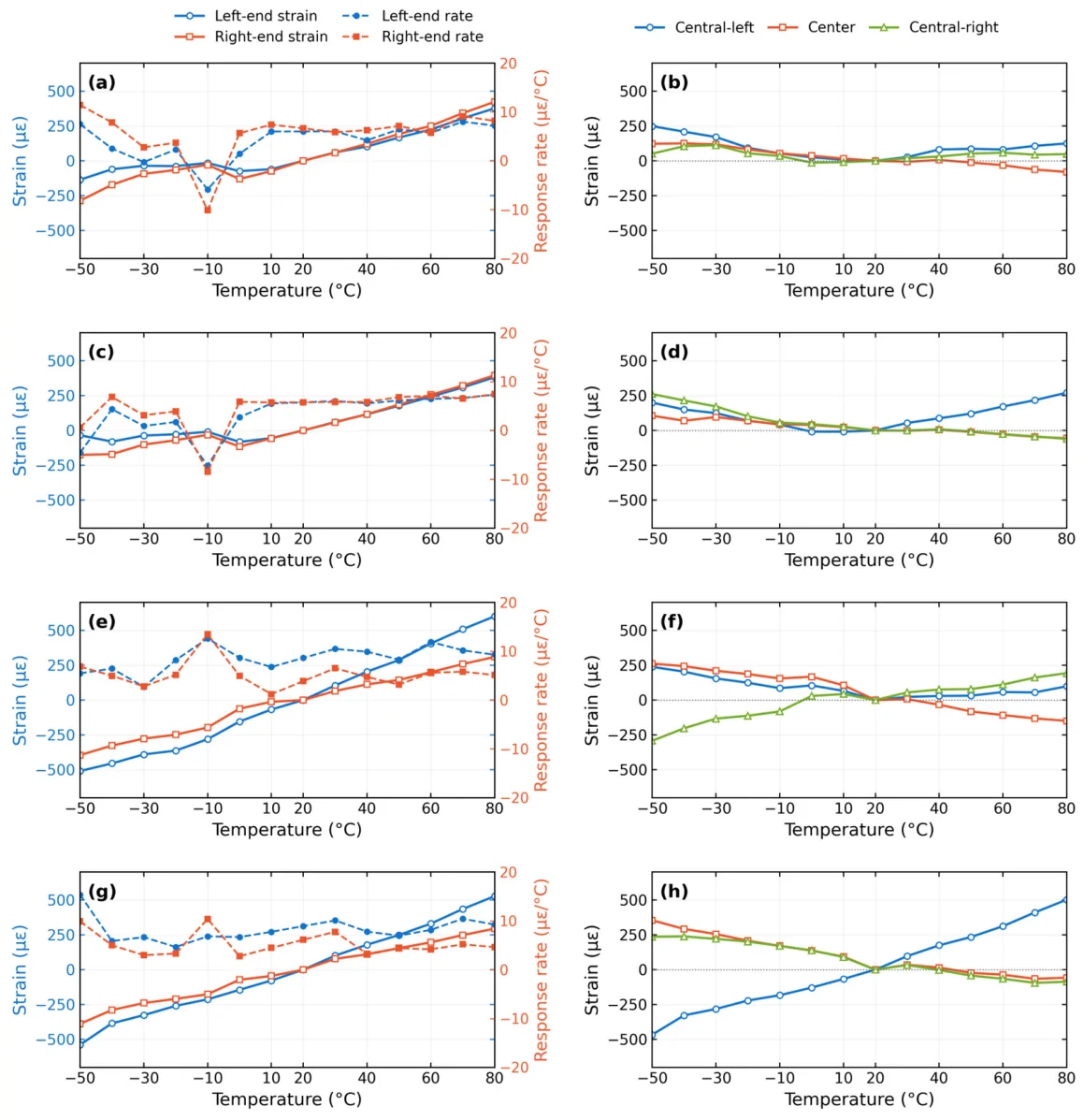
Regional evaluation of four couplers relative to the 20 °C reference: (**a**,**b**) 2 × 2 coupler C2500067439; (**c**,**d**) 2 × 2 coupler C2500068085; (**e**,**f**) 1 × 3 coupler C2500259001; and (**g**,**h**) 1 × 3 coupler C2500274905. The left-column panels show strain (solid curves, left *y*-axis) and adjacent-temperature response rate (dashed curves, right *y*-axis) at the two bonded transition regions. The right-column panels show strain at the central-left, center, and central-right positions within the fused-taper region.

Figure 11 applies the two regional screening criteria to four additional couplers: two 2 × 2 devices (C2500067439 and C2500068085) and two 1 × 3 devices (C2500259001 and C2500274905). The principal coupler and the four additional couplers were measured during separate experimental campaigns conducted under different room-temperature conditions. To reduce the possible influence of spatial-temperature non-uniformity on the reference measurement, the equilibrated room-temperature state of each campaign was used as the differential-strain reference. Accordingly, 25 °C was used for the principal coupler experiment, whereas 20 °C was used for the additional-coupler experiment. Both campaigns employed 10 °C temperature increments and the same equilibration and OFDR measurement procedures. Each dataset was analyzed as the strain change relative to its own reference state. Therefore, the regional trends and left–right differences were evaluated within each device, and absolute strain amplitudes were not directly compared between the two experimental campaigns. Each row represents one coupler. The left panels compare the strain and response rate between the two end-bonding regions, whereas the right panels compare strain at three central fused-taper region positions. Separation between the end-region curves was interpreted as package-bonding asymmetry, whereas separation among the central curves indicated non-uniform strain transfer through the fused-taper section. Because no quantitative separation threshold was specified, these assignments are comparative screening classifications rather than definitive diagnoses.

For C2500067439, Figure 11a indicates that the end-bonding asymmetry is concentrated mainly in the low-temperature range. From −50 to −30 °C, the strain difference between the two ends is 57.8–150.6 με, reaching its maximum of 150.6 με at −50 °C. The largest difference between the two response rates is 5.34 με/°C at −40 °C. Above 30 °C, the end-strain difference remains below 46.9 με. These results indicate that the main defect of this coupler is an unequal bonding constraint at the two ends, particularly under low-temperature contraction. Figure 11b shows that the three central-region curves remain relatively close from −20 to 30 °C, with a maximum spread of 39.5 με, except for 49.1 με at 0 °C. However, the spread increases to 197.0 με at −50 °C and 204.5 με at 80 °C. The fused-taper region is therefore comparatively uniform near the reference temperature but exhibits moderate non-uniformity at extreme temperatures. Overall, end-bonding asymmetry is the dominant defect in this coupler.

For C2500068085, Figure 11c shows that the two end-region curves are close over the range of 10–80 °C, where their strain difference does not exceed 14.3 με. The asymmetry becomes more evident below 0 °C: the difference increases from 42.8 με at −20 °C to 141.2 με at −50 °C, where the response rate difference also reaches its maximum of 5.15 με/°C. This indicates a low-temperature end-bonding asymmetry, although the package response is comparatively symmetric over most of the heating range. In Figure 11d, the spread among the three central-region curves is no more than 55.6 με from −20 to 30 °C. It then increases to 131.5 με at 50 °C, 200.2 με at 60 °C, 262.0 με at 70 °C, and 331.5 με at 80 °C. A similarly enlarged spread of 154.8 με occurs at −50 °C. The pronounced separation at high temperatures, especially at 80 °C, indicates clear non-uniformity in the central fused-taper region. Thus, fused-taper non-uniformity is the principal defect of this coupler.

For C2500259001, Figure 11e reveals end-bonding asymmetry over both the cooling and heating ranges. From −50 to −20 °C, the strain difference between the two ends remains between 113.1 and 127.7 με. Above 40 °C, it increases continuously from 91.2 με at 40 °C to 205.7 με at 60 °C and reaches 291.1 με at 80 °C. The maximum response rate difference is 6.32 με/°C at 60 °C. These results indicate persistent package-bonding non-uniformity, with the most serious degradation occurring at high temperatures. Figure 11f shows even stronger non-uniformity in the central fused-taper region. The spread among the three curves reaches 553.0 με at −50 °C and remains 298.6–445.3 με from −40 to −20 °C. It increases again above 40 °C, reaching 293.0 με at 70 °C and 342.1 με at 80 °C. The maximum degradation occurs at −50 °C, indicating severe fused-taper non-uniformity. This coupler therefore exhibits clear defects in both the end-bonding and central fused-taper regions.

For C2500274905, Figure 11g shows clear end-bonding asymmetry at both low and high temperatures. The end-strain difference is 149.7 με at −50 °C and remains between 49.9 and 95.4 με from −40 to −20 °C. During heating, the difference increases from 68.6 με at 40 °C to 134.1 με at 60 °C, 186.5 με at 70 °C, and a maximum of 232.7 με at 80 °C. The largest response rate difference is 5.43 με/°C at −50 °C. These results demonstrate asymmetric load transfer at the two bonding regions, with the most severe end-response degradation occurring at 80 °C. Figure 11h indicates pronounced non-uniformity throughout the central fused-taper region. The spread among the three curves reaches 819.6 με at −50 °C and remains between 428.2 and 619.2 με from −40 to −20 °C. It increases again from 177.4 με at 40 °C to 376.5 με at 60 °C and 587.9 με at 80 °C. The worst degradation occurs at −50 °C. Therefore, this coupler has both end-bonding asymmetry and severe fused-taper non-uniformity, with the latter being the more pronounced defect.

To verify the response-based evaluations directly, C2500067439 and C2500068085 were selected for dismantling. They represent two complementary cases: C2500067439 is dominated by an end-bonding indication with a comparatively uniform central response, whereas C2500068085 has comparatively symmetric end responses but a clearer central-region indication.

**Figure 12:**
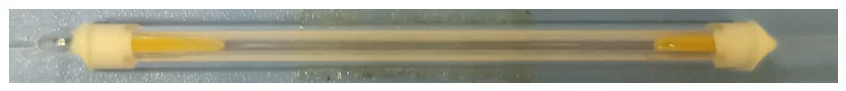
Dismantled end-bonding regions of 2 × 2 coupler C2500067439. The left end contains visibly more adhesive than the right end.

The unequal adhesive amount observed after dismantling agrees (Figure 12) with the end-response asymmetry in Figure 11a, while the comparatively regular central region in Figure 11b indicates that the principal abnormality of this sample is associated with the package-bonding regions.

**Figure 13:**
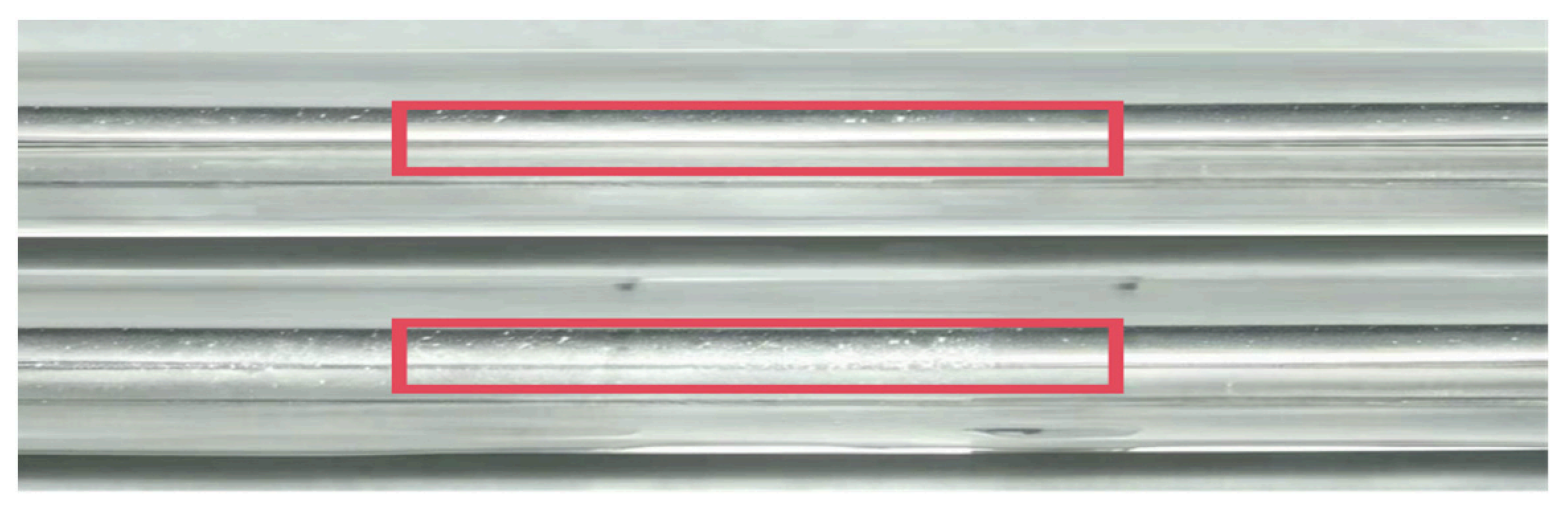
Dismantled fused-taper comparison. The central fused-taper region of C2500068085 (lower coupler) is visibly thinner than that of the upper comparison coupler.

The visibly thinner waist of C2500068085 (Figure 13) is consistent with the separated central-region responses in Figure 11d. At the same time, the relatively close end-region curves in Figure 11c indicate that its dominant abnormality is located in the fused-taper region rather than in the package-bonding regions.

The dismantling observations agree with the defect locations identified from the thermal-strain responses: unequal adhesive corresponds to end-bonding asymmetry, while an over-thin taper corresponds to central-region non-uniformity. Together with the consistent evaluation of both 2 × 2 and 1 × 3 devices in Figure 11, this agreement demonstrates the feasibility and general applicability of the proposed thermal-excitation OFDR method for screening the two principal coupler defect classes.

## 4. Discussion

This study evaluated the application of response analysis to detecting fused-taper non-uniformity and package-bonding non-uniformity in 2 × 2 and 1 × 3 couplers. These defects affect the optical coupling region and the primary thermomechanical load-transfer boundaries, respectively, and may lead to device performance degradation or failure. Owing to the limited scope of this paper, other process variations, including minor fiber misalignment, small internal voids, local curing differences, and residual assembly stress, will be further investigated in future work.

The method is most suitable for devices containing a continuous optical path compatible with OFDR interrogation, including suitable all-fiber and waveguide devices, but is not applicable to devices employing entirely free-space internal optical paths. It can detect only conditions that alter the thermally induced strain field transferred to an interrogable internal fiber. It can reveal spatial non-uniformity, asymmetric constraint, and localized load-transfer anomalies but cannot detect defects that produce no measurable thermal-strain contrast. Therefore, its primary role is comparative screening and defect localization rather than definitive causal diagnosis. When the physical cause must be determined, response analysis should be combined with independent inspection and, where appropriate, dismantling inspection.

Future validation should include couplers that meet relevant standards, specimens with independently documented variations in taper geometry and adhesive bonding, repeated heating and cooling cycles, and local temperature measurements at the specimen position. Post-test verification should employ quantitative microscopy or X-ray CT, and the sample size should be sufficient to estimate diagnostic thresholds and error rates.

## 5. Conclusions

We developed an OFDR-based method for characterizing internal defects in fiber couplers. The method employs active thermal excitation to nondestructively inspect packaged fiber couplers and evaluate spatial non-uniformity in their thermal-strain responses. A free reference fiber is used to acquire the temperature-response signal and subtract the contribution caused directly by temperature. A nominally symmetric finite-element model is then used to define the expected end-to-end symmetry and smoothness of the central-region response. In the primary 1 × 3 coupler experiment, the responses at the two end-bonding regions remained broadly similar over the investigated temperature range, providing no clear evidence of pronounced end-bonding non-uniformity in this specimen. Temperature-dependent spatial fluctuations were, however, observed within the central fused-taper window. In the four-coupler application validation, the same criteria were applied to compare the simulated and measured responses of both 2 × 2 and 1 × 3 configurations (Figure 11). The method therefore enables spatial localization and comparative screening of thermal-strain non-uniformity. Future work will expand the sample size and device types, introduce reference couplers with independently documented taper and bonding conditions, and perform repeated heating–cooling tests with local temperature measurements. Quantitative microscopy or X-ray CT will also be used to verify the detected abnormalities, establish screening thresholds, and further evaluate the applicability of the method to other packaged fiber-optic devices.

## Figures and Tables

**Table 1:** The material parameters of the coupler.

Parameter	SIL	EP	UV-Curable	EP-Sealant	Fiber
E(10^9^ Pa)	0.005	0.18	0.011	0.01	80
CTE × 10^−6^ (1/K)	450	200	112.5	630	0.55 [19]
Poisson’s ratio	0.4	0.26	0.15	0.16	0.22
Density (g/cm^3^)	1	1.2	1.25	1.2	2.203

**Table 2:** Quantitative comparison of simulated and experimental strain profiles at ten non-reference temperatures. All errors were calculated without fitted spatial or amplitude alignment.

T (°C)	RMSE (µε)	NRMSE (%)	Pearson r	Peak Pos. Error (mm)	Exp. Peak (µε)	Sim. Peak (µε)	Peak Amp. Error (µε; %)
35	10.36	9.29	0.974	0.27	111.54	101.21	10.33; 9.26%
45	18.04	8.39	0.978	0.27	215.11	202.43	12.69; 5.90%
55	42.43	14.63	0.936	0.55	290.05	276.50	13.55; 4.67%
65	47.36	12.42	0.957	0.27	381.47	368.66	12.81; 3.36%
15	26.21	26.42	0.970	0.15	−99.21	−135.69	36.48; 36.77%
5	77.20	37.21	0.978	0.15	−207.45	−271.39	63.94; 30.82%
−5	39.38	12.97	0.982	0.24	−303.67	−305.00	1.33; 0.44%
−15	57.14	14.10	0.977	0.42	−405.36	−406.43	1.07; 0.26%
−25	81.85	16.39	0.965	0.45	−499.27	−501.10	1.83; 0.37%
−35	112.39	19.36	0.950	0.46	−580.46	−601.13	20.67; 3.56%

## Data Availability

The experimental and simulation data generated are available from the corresponding author upon reasonable request.
